# Investigating the associations between dietary nutrient intake and risk of Hashimoto’s thyroiditis: a cross-sectional study from NHANES and a case-control study

**DOI:** 10.3389/fnut.2025.1731662

**Published:** 2025-12-31

**Authors:** Yicheng Wang, Lina Zhang, Xinqi Wang, Xiangyi Yuan, Qingling Huang, Hao Wang, Qingyuan Song, Duo Li, Hui Kan, Jiaomei Li

**Affiliations:** 1 School of Public Health, Zhejiang Chinese Medical University, Hangzhou, China; 2 Dongying Center for Disease Control and Prevention, Dongying, China; 3 Institute of Nutrition and Health, Qingdao University, Qingdao, China; 4 School of Stomatology, Zhejiang Chinese Medical University, Hangzhou, China

**Keywords:** dietary nutrients, Hashimoto’s thyroiditis, n-3 PUFAs, NHANES, polyunsaturated fatty acids

## Abstract

**Background:**

Hashimoto’s thyroiditis (HT) is a prevalent autoimmune disorder and a leading cause of hypothyroidism, with an increasing incidence worldwide. Emerging evidence suggests that dietary nutrients intake may influence the development and progression of HT.

**Methods:**

Data from the 2009–2012 National Health and Nutrition Examination Survey (NHANES) were analyzed to examine associations between dietary nutrient intake and HT risk among 2,732 participants. Nutrient intakes were categorized into quartiles, with the lowest quartile (Q1) serving as the reference. Weighted multivariable logistic regression models were applied to assess associations, and restricted cubic splines models were used to evaluate potential nonlinear relationships. To validate the NHANES findings and reduce potential biases arising from dietary assessment inaccuracies and racial differences, a case-control study was conducted to investigate the association between erythrocyte phospholipid fatty acids and HT risk using logistic regression models.

**Results:**

No significant correlations were found between HT risk and the intake of carbohydrates, sugars, fiber, vitamin C, vitamin D, vitamin E, magnesium, or zinc (*p* > 0.05). In contrast, higher total polyunsaturated fatty acids (PUFAs) and *α*-linolenic acid (C18:3 n-3) intake were significantly associated with lower HT risk (*p* < 0.05), although no nonlinear relationships were observed. Subgroup analysis revealed that α-linolenic acid intake was significantly lower in females than in males, potentially contributing to their greater susceptibility to HT. In the validation study, higher erythrocyte membrane levels of total n-3 PUFAs (OR = 0.616, 95% CI: 0.443–0.992, *p* = 0.041), C18:3 n-3 (OR = 0.627, 95% CI: 0.558–0.874, *p* = 0.037) and eicosapentaenoic acid (EPA, C20:5 n-3; OR = 0.736, 95% CI: 0.581–0.975, *p* = 0.042) were significantly associated with a low prevalence of HT.

**Conclusion:**

The findings suggest that insufficient n-3 PUFAs intake may be a potential risk factor of Hashimoto’s thyroiditis. Clinical trials are warranted to evaluate whether n-3 PUFA supplementation could help prevent or mitigate thyroid autoimmunity.

## 1 Introduction

Hashimoto’s thyroiditis (HT), a prevalent autoimmune disorder, is the leading cause of hypothyroidism. It is characterized by lymphocytic infiltration of the thyroid gland and the production of thyroid-specific autoantibodies, particularly anti-thyroid peroxidase (TPOAb) and anti-thyroglobulin (TgAb) antibodies (1). Epidemiological data indicate a steady rise in HT prevalence in recent years, with a pronounced female predominance (2, 3). Among laboratory diagnostic markers, TPOAb demonstrates greater sensitivity and specificity than TgAb, making it the preferred biomarker for HT diagnosis (4). Elevated TPOAb levels have been associated with several adverse clinical outcomes. For instance, a meta-analysis reported that TPOAb-positive pregnant women had a threefold increased risk of miscarriage and a twofold higher risk of preterm delivery (5). Moreover, TPOAb positivity has been linked to an increased risk of postpartum depression and is biologically associated with depressive disorders even in euthyroid individuals (6, 7). Currently, there are no effective therapeutic strategies for reducing TPOAb levels, underscoring the need for effective preventive measures to reduce HT incidence.

The pathogenesis of HT involves a complex interplay between genetic predisposition and environmental factors (8). Although genetic factors account for approximately 80% of HT susceptibility (9), modifiable environmental elements, especially dietary patterns and nutrient intake, play a critical role in disease onset and progression. Recent studies suggest that long-term adherence to a gluten-free diet may improve thyroid function by mitigating inflammation and dampening immune system overactivation (10). Nutritional imbalances such as selenium deficiency, which exacerbates Th1/Th2 immune imbalance and autoimmunity, and excessive iodine intake, which triggers oxidative stress and lymphocytic infiltration of the thyroid, have also been implicated in autoimmune thyroid diseases (11, 12). In addition, magnesium and zinc are involved in thyroid hormone synthesis, antioxidant defense, and immune regulation (13). While certain nutrients have been associated with HT pathogenesis, many dietary components remain insufficiently explored.

The National Health and Nutrition Examination Survey (NHANES), conducted by the National Center for Health Statistics (NCHS), provides nationally representative data through a stratified, multistage probability sampling design. Recent analyses of NHANES data have identified positive correlations between specific saturated fatty acids (e.g., C10:0, C12:0, C14:0) and serum TPOAb levels, suggesting that dietary lipid profiles may influence thyroid immune homeostasis (14).

In this study, we aimed to systematically investigate the association between dietary nutrient intake and the risk of HT using data from NHANES 2009–2012. Additionally, to validate these findings and address limitations inherent to dietary self-reporting and population heterogeneity, we conducted a complementary case-control study. This research may provide a scientific basis for the development of dietary interventions aimed at preventing HT.

## 2 Materials and methods

### 2.1 Study population from the NHANES database

**Figure 1:**
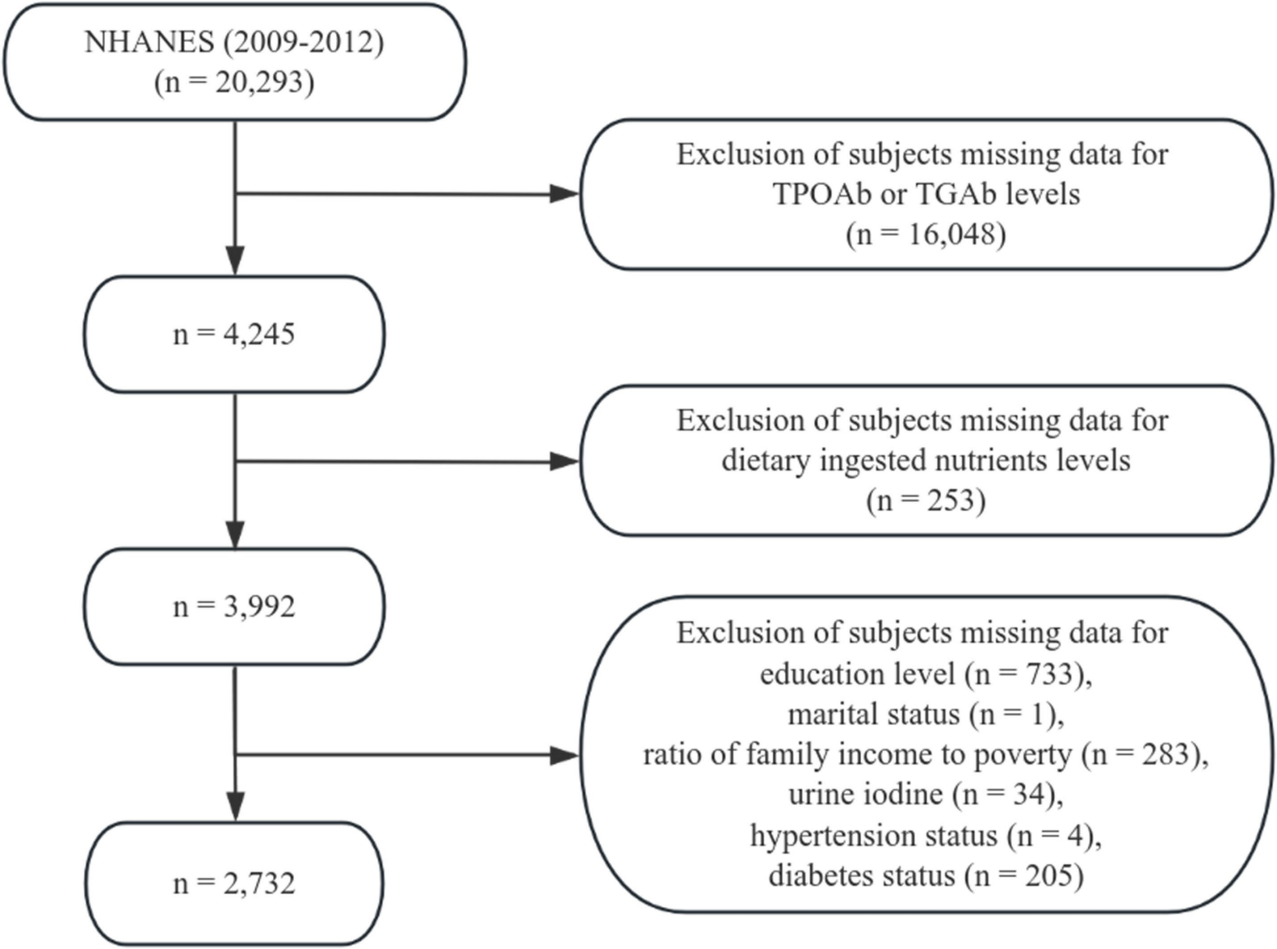
Flowchart of participant selection from the 2009–2012 National Health and Nutrition Examination Survey (NHANES).

This study utilized demographic information, laboratory data, and dietary questionnaire data from the NHANES for the years 2009–2012. A total of 20,293 participants who had completed dietary interviews, medical examinations, and health questionnaires were initially considered. The following exclusion criteria were applied: (1) missing data for TPOAb or TGAb levels (*n* = 16,048); (2) missing data for dietary ingested nutrients (*n* = 253); (3) missing education level (*n* = 733); (4) missing marital status (*n* = 1); (5) missing data on the family income-to-poverty ratio (Federal Poverty Level, FPL) (*n* = 283); (6) missing data for urine iodine (*n* = 34); (7) missing data for hypertension status (*n* = 4); and (8) missing data for diabetes status (*n* = 205). After exclusions, 2,732 participants were included in the final analysis (Figure 1).

### 2.2 Assessment of Hashimoto’s thyroiditis

TPOAb and TGAb levels were obtained from NHANES laboratory data. TPOAb and TGAb levels were measured using the Access immunoassay system (Beckman Coulter), which employs a sequential two-step sandwich chemiluminescent assay. According to the manufacturer’s guidelines, participants were classified as Hashimoto’s thyroiditis-positive if TPOAb > 9 IU/mL or TGAb > 4 IU/mL.

### 2.3 Dietary nutrient intake assessment

Dietary nutrient intake was evaluated based on 24-h dietary recall data collected on the first day of the NHANES dietary interview. Nutrient variables included energy, total fat, total polyunsaturated fatty acids (PUFAs), protein, carbohydrates, total sugars, dietary fiber, vitamin C, vitamin D (D2 and D3), vitamin E (as alpha-tocopherol), magnesium, and zinc. PUFA subtypes included C18:2 n-6, C18:3 n-3, C18:4 n-3, C20:4 n-6, C20:5 n-3, C22:5 n-3, and C22:6 n-3.

### 2.4 Covariates

The following variables were considered potential confounders and were included as covariates in the regression models: sex, age, race, education level, marital status, ratio of family income to poverty, smoking status, total cholesterol, direct HDL-cholesterol, selenium, urinary iodine and alcohol consumption status. Age was categorized as 20 ~ 30, 30 ~ 50, and > 50 years. Race was classified as Mexican American, other Hispanics, non-Hispanic White, non-Hispanic Black, and other (including multiracial). Education level was grouped into four categories: less than 11th grade (including 12th grade without diploma), high school graduate/general educational development (GED), some college or associate degree, and college graduate or above. Marital status was divided into married or living with partner, divorced or separated and never married. FPL was categorized as ≤ 130, 130% ~ 350%, and > 350%. Smoking status was divided into current, former, and never smokers. Alcohol consumption status was divided into heavy, light, and never drinkers.

### 2.5 Population of the case-control study

The sample size calculation was based on the formula: *n* = (*Z*^2^ × *p* × (1- *p*)) /*e*^2^, where *Z* corresponds to the standard normal deviate at the desired confidence level, *p* is the estimated prevalence, and *e* denotes the margin of error (15). For a 95% confidence level, *Z* was set to 1.96. According +to previous studies, the prevalence of HT is about 7.5% (16). Substituting *p* = 0.12 and *e* = 0.05 into the formula yielded a minimum required sample size of 107 patients with HT. Healthy subjects were then matched to HT cases at a 1:2 ratio, resulting in at least 54 controls.

A total of 125 HT patients and 57 healthy controls were recruited from two hospitals: 51 HT patients and 22 controls from the Affiliated Hospital of Qingdao University, and 74 HT patients and 35 controls from Zhejiang Chinese Medical University. HT patients were eligible if they were adults aged 18–60 years who had been clinically confirmed by an endocrinologist based on typical ultrasonographic features together with positive thyroid autoantibodies. Individuals in the control group were selected from routine health-check programs at the same institutions and were frequency-matched to cases by age and sex. Only participants without any history of thyroid disorders, including functional abnormalities or nodular disease, were considered as controls. General exclusion criteria were applied to both groups, including pregnancy or breastfeeding, a history of alcohol dependence, concomitant autoimmune conditions (e.g., rheumatoid arthritis, multiple sclerosis), severe chronic illnesses such as malignancies or cardiovascular disease, and recent use of immunosuppressive medications or n-3 PUFA supplementation. Demographic characteristics and lifestyle information were collected through structured interviews by trained investigators. All participants signed written informed consent prior to enrollment. The study protocol was approved by the Ethics Committee of Qingdao University (approval number: QDU-20201107-1). All procedures were following institutional ethical guidelines and the Declaration of Helsinki.

### 2.6 Blood sample collection and fatty acid composition analysis

Following a 12-h overnight fast, venous blood samples were collected for lipid analysis. Samples were centrifuged at 1500 × g for 15 min at 4 °C to separate plasma and red blood cells (RBCs), which were subsequently stored at −80 °C until analysis. Total lipids were extracted from 0.5 mL of RBCs using a methanol: chloroform solution (2:1, v/v). The phospholipid fraction was isolated by thin-layer chromatography and transmethylated using methanol containing sulfuric acid (2%, v/v) at 70 °C for 2 h. After cooling to room temperature, the resulting fatty acid methyl esters (FAMEs) were extracted with hexane, dried under nitrogen, and redissolved for analysis by gas chromatography (GC-2014 system, Shimadzu) equipped with a flame ionization detector and an AOC-20i auto-injector. A DB-23 capillary column (60 m × 0.25 mm internal diameter × 0.15 μm film thickness, Agilent Technologies, USA) was used for separation. Individual fatty acids were identified by comparing retention times with a commercial standard mixture containing 32 FAMEs. Results were expressed as the relative percentage of each fatty acid out of the total fatty acids (17). The n-3 index was calculated as the combined percentage of eicosapentaenoic acid (EPA) and docosahexaenoic acid (DHA).

### 2.7 Statistical analyses

NHANES uses a complex, multistage probability sampling design. To account for this, we applied sampling weights (WTDRD1), primary sampling units (SDMVPSU), and strata (SDMVSTRA) as recommended in the NHANES analytic guidelines. Continuous variables were presented as weighted means and standard deviations (SDs), while categorical variables were expressed as weighted percentages (%).

Dietary nutrient intake levels were categorized into quartiles, with the lowest quartile (Q1) serving as the reference group. Weighted multivariable logistic regression models were used to determine the associations between dietary nutrient intake and the risk of HT. Three models were constructed: Model 1, unadjusted; Model 2, adjusted for sex, age, race, education level, marital status, and ratio of family income to poverty; Model 3, additionally adjusted for smoking status, total cholesterol, direct HDL-cholesterol, selenium, urinary iodine, and alcohol consumption status. Additionally, sensitivity analyses were performed by further adjusting for hypertension and diabetes to examine the robustness of the conclusions from Model 3. The nonlinear associations of total PUFAs intake and C18:3 n-3 intake with the risk of HT were assessed using restricted cubic splines (RCS).

In the case-control study, continuous variables are expressed as mean and SD, and categorical variables as percentages. Logistic regression models were used to determine the associations between erythrocyte phospholipid PUFA levels and HT status. Model 1, unadjusted; Model 2, adjusted for age and sex; Model 3, further adjusted for marital status, family history of HT, body mass index, educational level, total erythrocyte n-6 PUFA, and total saturated fatty acids. A two-tailed *p*-value less than 0.05 was considered statistically significant. All statistical analyses were performed using R software (version 4.3.2).

## 3 Results

### 3.1 Demographic and clinical characteristics of NHANES participants

After applying inclusion and exclusion criteria, a total of 2,732 participants were included in this study. Among them, 1,333 (52%) were female, 1,255 (42%) were over 50 years of age, 1,262 (70%) were non-Hispanic White, 643 (30%) had attained a college degree or higher, 1,608 (62%) were married or living with partner, 835 (42%) had a higher income level, 963 (31%) had hypertension, 327 (9.4%) had diabetes, 1,486 (55%) were never smokers, and 2,033 (80%) were heavy drinkers. Statistically significant differences were observed between the HT-positive and HT-negative groups in sex, age, race and total cholesterol levels (*p* < 0.05; Table 1).

**Table 1:** Weighted demographic and clinical characteristics of NHANES participants based on Hashimoto’s thyroiditis.

Characteristic	Overall *N* = 2,732 (100%)^1^	Hashimoto’s thyroiditis		*p* value^2^
		Negative	Positive	
		(TPOAb ≤ 9 IU/mL and TGAb ≤ 4 IU/mL)	(TPOAb > 9 IU/mL or TGAb > 4 IU/mL)	
		*N* = 2,341 (83%)^1^	*N* = 391 (17%)^1^	
Sex (%)				**< 0.001**
Male	1,399 (48%)	1,246 (51%)	153 (36%)	
Female	1,333 (52%)	1,095 (49%)	238 (64%)	
Age (%)				**0.003**
20 ~ 30y	557 (22%)	511 (23%)	46 (13%)	
30 ~ 50y	920 (37%)	801 (37%)	119 (35%)	
>50y	1,255 (42%)	1,029 (40%)	226 (52%)	
Race (%)				**0.005**
Mexican American	381 (7.6%)	334 (8.0%)	47 (5.1%)	
Other Hispanic	257 (5.5%)	214 (5.3%)	43 (6.4%)	
Non-Hispanic White	1,262 (70%)	1,042 (69%)	220 (78%)	
Non-Hispanic Black	565 (10.0%)	523 (11%)	42 (3.9%)	
Other race - including multi-racial	267 (6.5%)	228 (6.4%)	39 (7.1%)	
Education level (%)				0.126
Less than 11th grade (Includes 12th grade with no diploma)	659 (15%)	579 (16%)	80 (11%)	
High school graduate/GED or equivalent	602 (22%)	515 (22%)	87 (23%)	
Some college or AA degree	828 (32%)	707 (33%)	121 (30%)	
College graduate or above	643 (30%)	540 (29%)	103 (35%)	
Marital status (%)				0.519
Married or living with partner	1,608 (62%)	1,388 (62%)	220 (59%)	
Divorced or separated	596 (18%)	486 (17%)	110 (21%)	
Never married	528 (20%)	467 (21%)	61 (20%)	
Ratio of family income to poverty (%)				0.191
≤130%	884 (23%)	773 (23%)	111 (19%)	
130% ~ 350%	1,013 (35%)	866 (36%)	147 (34%)	
>350%	835 (42%)	702 (41%)	133 (48%)	
Total cholesterol (mmol/L)	5.096 (1.087)	5.065 (1.073)	5.252 (1.145)	0.052
Direct HDL-cholesterol (mmol/L)	1.379 (0.420)	1.367 (0.418)	1.439 (0.426)	**0.031**
Selenium (%)	111.086 (59.667)	112.883 (60.854)	102.006 (52.399)	**0.019**
Urine iodine (ug/L)	224.380 (841.028)	230.157 (903.543)	195.192 (394.787)	0.996
Hypertension status (%)				0.775
Yes	963 (31%)	813 (31%)	150 (32%)	
No	1,769 (69%)	1,528 (69%)	241 (68%)	
Diabetes status (%)				0.060
Yes	327 (9.4%)	268 (8.8%)	59 (12%)	
No	2,347 (89%)	2,022 (89%)	325 (87%)	
Borderline	58 (1.8%)	51 (2.0%)	7 (1.1%)	
Smoking status (%)				0.277
Current smokers	543 (21%)	490 (22%)	53 (16%)	
Former smokers	703 (24%)	597 (24%)	106 (25%)	
Never smokers	1,486 (55%)	1,254 (54%)	232 (59%)	
Alcohol consumption status (%)				0.774
Heavy drinkers	2,033 (80%)	1,763 (80%)	270 (78%)	
Light drinkers	345 (11%)	285 (11%)	60 (12%)	
Never drinkers	354 (9.1%)	293 (9.1%)	61 (9.5%)	

1 mean (sd) for continuous; *n*, unweighted (%-weighted) for categorical.

2 Pearson’s X^2^: Rao & Scott adjustment; Design-based Kruskal-Wallis test.

Bold values represent statistical significance.

TPOAb, anti-thyroid peroxidase; TGAb, anti-thyroglobulin.

Univariate analysis showed that females had a significantly higher risk of HT compared to males (OR = 1.862, 95% CI: 1.368–2.535, *p* < 0.001). With individuals aged 20–30 years as the reference, those over 50 years (OR = 2.323, 95% CI: 1.437–3.755, *p* = 0.001) exhibited significantly increased HT risk (Table 2).

**Table 2:** Univariate analysis of the associations between sex, age, race, and the risk of Hashimoto’s thyroiditis.

Characteristic	OR (95%CI)	*p* value^1^
Sex (%)		
Male	Reference	
Female	1.862 (1.368, 2.535)	**<0.001**
Age (%)		
20 ~ 30y	Reference	
30 ~ 50y	1.711 (0.972, 3.012)	0.062
>50y	2.323 (1.437, 3.755)	**0.001**
Race (%)		
Mexican American	Reference	
Other Hispanic	1.910 (0.851,4.285)	0.112
Non-Hispanic White	1.752 (0.992·,3.094)	0.053
Non-Hispanic Black	0.536 (0.277,1.035)	0.063
Other race - including multi-racial	1.709 (0.730,4.001)	0.208

1 Logistic regression models: no covariates were adjusted in univariate analysis.

Bold values represent statistical significance.

OR, odds ratio; CI, confidence interval.

### 3.2 Dietary nutrient intake levels among NHANES participants

Compared to the HT-negative group, energy (1,889.667 ± 753.040 kcal vs. 2,212.200 ± 1,051.583 g, *p* < 0.001) and total fat (67.181 ± 36.684 g vs. 82.990 ± 47.093 g, *p* < 0.001) were significantly lower in the HT-positive group. Total PUFA intake was also significantly lower in the HT-positive group (16.007 ± 10.276 g vs. 19.248 ± 12.686 g, *p* = 0.002). Specifically, intakes of linoleic acid (C18:2 n-6; 14.195 ± 9.444 g vs. 17.065 ± 11.504 g, *p* = 0.002), *α*-linolenic acid (C18:3 n-3; 1.417 ± 0.942 g vs. 1.701 ± 1.192 g, *p* = 0.009), arachidonic acid (C20:4 n-6; 0.117 ± 0.112 g vs. 0.153 ± 0.146 g, *p* = 0.004), and docosapentaenoic acid (DPA, C22:5 n-3; 0.018 ± 0.030 g vs. 0.025 ± 0.051 g, *p* < 0.001) were all significantly lower among HT patients. Protein (74.376 ± 34.562 g vs. 83.987 ± 42.939 g, *p* = 0.004), carbohydrate (232.981 ± 97.791 g vs. 264.785 ± 131.062 g, *p* = 0.003), and total sugars intake (98.785 ± 60.853 g vs. 118.017 ± 80.378 g, *p* = 0.002) were also significantly lower in the HT-positive group. Eicosapentaenoic acid (C20:5 n-3; 0.038 ± 0.130 g vs. 0.030 ± 0.094 g, *p* = 0.014) and vitamins C (89.934 ± 80.980 g vs. 81.647 ± 81.647 g, *p* = 0.042) were significantly higher in the HT-positive group. No significant differences were observed for C18:4 n-3, C22:6 n-3, dietary fiber, vitamins D, E, magnesium, or zinc (all *p* > 0.05; Table 3).

**Table 3:** Weighted dietary nutrient intake levels among NHANES participants based on Hashimoto’s thyroiditis.

Characteristic	Overall *N* = 2,732 (100%)^1^	Hashimoto’s thyroiditis		*p* value^2^
		Negative	Positive	
		(TPOAb ≤ 9 IU/mL and TGAb ≤ 4 IU/mL)	(TPOAb > 9 IU/mL or TGAb > 4 IU/mL)	
		*N* = 2,341 (83%)^1^	*N* = 391 (17%)^1^	
Energy (kcal)	2,158.911 (1,015.335)	2,212.200 (1,051.583)	1,889.667 (753.040)	**<0.001**
Total fat (gm)	80.378 (45.908)	82.990 (47.093)	67.181 (36.684)	**<0.001**
Total PUFAs (gm)	18.712 (12.377)	19.248 (12.686)	16.007 (10.276)	**0.002**
C18:2 n-6 (gm)	16.591 (11.239)	17.065 (11.504)	14.195 (9.444)	**0.002**
C18:3 n-3 (gm)	1.654 (1.159)	1.701 (1.192)	1.417 (0.942)	**0.009**
C18:4 n-3 (gm)	0.012 (0.034)	0.012 (0.034)	0.012 (0.036)	0.437
C20:4 n-6 (gm)	0.147 (0.142)	0.153 (0.146)	0.117 (0.112)	**0.004**
C20:5 n-3 (gm)	0.032 (0.101)	0.030 (0.094)	0.038 (0.130)	**0.014**
C22:5 n-3 (gm)	0.024 (0.048)	0.025 (0.051)	0.018 (0.030)	**<0.001**
C22:6 n-3 (gm)	0.065 (0.177)	0.063 (0.172)	0.071 (0.200)	0.247
Protein (gm)	82.399 (41.818)	83.987 (42.939)	74.376 (34.562)	**0.004**
Carbohydrate (gm)	259.530 (126.706)	264.785 (131.062)	232.981 (97.791)	**0.003**
Total sugars (gm)	114.840 (77.810)	118.017 (80.378)	98.785 (60.853)	**0.002**
Dietary fiber (gm)	17.311 (10.412)	17.282 (10.481)	17.460 (10.066)	0.778
Vitamin C (mg)	83.016 (90.157)	81.647 (91.821)	89.934 (80.980)	**0.042**
Vitamin D (D2 + D3) (mcg)	4.857 (5.560)	4.887 (5.655)	4.704 (5.055)	0.937
Vitamin E as alpha-tocopherol (mg)	8.313 (6.259)	8.366 (6.298)	8.046 (6.055)	0.077
Magnesium (mg)	308.025 (151.896)	310.911 (155.762)	293.443 (129.814)	0.295
Zinc (mg)	11.549 (6.872)	11.664 (6.746)	10.970 (7.458)	0.077

1 mean (sd) for continuous; *n* -unweighted (%-weighted) for categorical.

2 Pearson’s X^2^: Rao & Scott adjustment; Design-based Kruskal-Wallis test.

Bold values represent statistical significance.

TPOAb, anti-thyroid peroxidase; TGAb, anti-thyroglobulin; PUFA, polyunsaturated fatty acid.

### 3.3 Association between dietary nutrient intake and HT risk

Associations between dietary nutrient intake and the risk of HT are shown in Table 4. In the unadjusted model (Model 1), higher intake of energy, total fat, total PUFAs, C18:2 n-6, C18:3 n-3, C20:4 n-6, protein, carbohydrates, and total sugars were all associated with decreased risk of HT. However, after adjustment for covariates in Models 2 and 3, only energy, total fat, total PUFAs and C18:3 n-3 remained significantly associated with lower HT risk (*p* < 0.05).

**Table 4:** Associations between dietary nutrient intake levels and the risk of Hashimoto’s thyroiditis.

Characteristic	Model 1		Model 2		Model 3	
	OR (95% CI)	*p* value	OR (95% CI)	*p* value	OR (95% CI)	*p* value
Energy (kcal)	0.99962 (0.99948, 0.99977)	**<0.001**	0.99975 (0.99961, 0.99988)	**0.001**	0.99965 (0.99934, 0.99996)	**0.029**
Quartile						
Q1	Reference		Reference		Reference	
Q2	0.788 (0.528, 1.175)	0.233	0.768 (0.495, 1.190)	0.219	0.745 (0.464, 1.197)	0.190
Q3	0.782 (0.535, 1.143)	0.195	0.890 (0.586, 0.586)	0.565	0.836 (0.493, 1.416)	0.455
Q4	0.396 (0.267, 0.587)	**<0.001**	0.536 (0.364, 0.789)	**0.003**	0.486 (0.242, 0.973)	**0.043**
Total fat (gm)	0.991 (0.987, 0.995)	**<0.001**	0.993 (0.989, 0.996)	**<0.001**	0.990 (0.985, 0.996)	**0.003**
Quartile						
Q1	Reference		Reference		Reference	
Q2	0.606 (0.401, 0.914)	**0.019**	0.599 (0.393, 0.915)	**0.021**	0.585 (0.361, 0.950)	**0.034**
Q3	0.490 (0.303, 0.792)	**0.005**	0.510 (0.304, 0.856)	**0.014**	0.473 (0.257, 0.872)	**0.022**
Q4	0.352 (0.229, 0.541)	**<0.001**	0.435 (0.291, 0.650)	**<0.001**	0.371 (0.209, 0.658)	**0.004**
Total PUFAs (gm)	0.975 (0.960, 0.990)	**0.002**	0.980 (0.965, 0.996)	**0.016**	0.978 (0.957, 1.000)	**0.049**
Quartile						
Q1	Reference		Reference		Reference	
Q2	0.644 (0.4031.030)	0.065	0.642 (0.406, 1.015)	0.057	0.643 (0.378, 1.096)	0.093
Q3	0.627 (0.379, 1.037)	0.068	0.655 (0.391, 1.097)	0.101	0.638 (0.353, 1.153)	0.118
Q4	0.445 (0.268, 0.738)	**0.003**	0.519 (0.311, 0.866)	**0.015**	0.498 (0.247, 1.006)	0.052
C18:2 n-6 (gm)	0.973 (0.956, 0.991)	**0.004**	0.979 (0.962, 0.997)	**0.022**	0.978 (0.954, 1.002)	0.064
Quartile						
Q1	Reference		Reference		Reference	
Q2	0.607 (0.361, 1.020)	0.059	0.608 (0.364, 1.014)	0.056	0.609 (0.336, 1.104)	0.091
Q3	0.662 (0.402, 1.090)	0.102	0.667 (0.404, 1.100)	0.105	0.656 (0.367, 1.172)	0.132
Q4	0.453 (0.277, 0.741)	**0.003**	0.536 (0.327, 0.877)	**0.016**	0.519 (0.265, 1.016)	0.054
C18:3 n-3 (gm)	0.779 (0.671, 0.903)	**0.002**	0.802 (0.684, 0.941)	**0.009**	0.786 (0.642, 0.963)	**0.025**
Quartile						
Q1	Reference		Reference		Reference	
Q2	0.748 (0.471, 1.189)	0.210	0.738 (0.453, 1.202)	0.205	0.741 (0.434, 1.266)	0.233
Q3	0.813 (0.482, 1.372)	0.425	0.838 (0.491, 1.430)	0.493	0.841 (0.446, 1.587)	0.233
Q4	0.496 (0.306, 0.803)	**0.006**	0.523 (0.312, 0.874)	**0.017**	0.507 (0.265, 0.969)	**0.042**
C18:4 n-3 (gm)	0.861 (0.008, 92.244)	0.948	1.695 (0.009, 327.041)	0.835	3.288 (0.025, 436.780)	0.599
Quartile						
Q1	Reference		Reference		Reference	
Q2	0.878 (0.530, 1.455)	0.603	0.901 (0.552, 1.470)	0.657	0.897 (0.528, 1.523)	0.649
Q3	0.766 (0.490, 1.199)	0.234	0.822 (0.518, 1.304)	0.381	0.866 (0.537, 1.395)	0.505
Q4	0.919 (0.582, 1.451)	0.707	1.084 (0.658, 1.785)	0.737	1.167 (0.702, 1.941)	0.504
C20:4 n-6 (gm)	0.103 (0.022, 0.484)	**0.005**	0.226 (0.052, 0.974)	**0.046**	0.178 (0.028, 1.132)	0.064
Quartile						
Q1	Reference		Reference		Reference	
Q2	0.717 (0.430, 1.196)	0.195	0.770 (0.452, 1.309)	0.312	0.755 (0.406, 1.406)	0.328
Q3	0.546 (0.332, 0.899)	**0.019**	0.629 (0.364, 1.086)	0.091	0.618 (0.333, 1.147)	0.111
Q4	0.484 (0.295, 0.793)	**0.005**	0.629 (0.381, 1.040)	0.068	0.599 (0.315, 1.138)	0.103
C20:5 n-3 (gm)	1.918 (0.468, 7.856)	0.354	1.910 (0.391, 9.337)	0.403	2.467 (0.486, 12.530)	0.244
Quartile						
Q1	Reference		Reference		Reference	
Q2	0.876 (0.503, 1.527)	0.631	0.934 (0.521, 1.676)	0.808	0.932 (0.493, 1.762)	0.804
Q3	0.590 (0.353, 0.985)	**0.044**	0.674 (0.401, 1.132)	0.126	0.682 (0.389, 1.198)	0.156
Q4	0.646 (0.425, 0.982)	**0.041**	0.740 (0.463, 1.182)	0.192	0.741 (0.434, 1.264)	0.232
C22:5 n-3 (gm)	0.001 (0.000, 4.998)	0.107	0.020 (0.000, 13.806)	0.225	0.033 (0.000, 28.695)	0.289
Quartile						
Q1	Reference		Reference		Reference	
Q2	0.857 (0.857, 1.318)	0.470	0.911 (0.560, 1.483)	0.691	0.903 (0.525, 1.553)	0.676
Q3	0.535 (0.329, 0.871)	**0.014**	0.601 (0.356, 1.017)	0.057	0.598 (0.333, 1.072)	0.077
Q4	0.471 (0.301, 0.739)	**0.002**	0.614 (0.377, 0.998)	**0.049**	0.597 (0.327, 1.089)	0.083
C22:6 n-3 (gm)	1.255 (0.565, 2.787)	0.566	1.241 (0.482, 3.192)	0.638	1.454 (0.542, 3.902)	0.417
Quartile						
Q1	Reference		Reference		Reference	
Q2	0.630 (0.399, 0.995)	**0.048**	0.643 (0.396, 1.046)	0.072	0.639 (0.381, 1.074)	0.082
Q3	0.624 (0.365, 1.067)	0.083	0.675 (0.386, 1.179)	0.155	0.684 (0.373, 1.253)	0.186
Q4	0.755 (0.496, 1.149)	0.182	0.804 (0.491, 1.315)	0.361	0.824 (0.469, 1.447)	0.451
Protein (gm)	0.994 (0.990, 0.997)	**<0.001**	0.997 (0.993, 1.000)	0.072	0.993 (0.980, 1.006)	0.271
Quartile						
Q1	Reference		Reference		Reference	
Q2	0.950 (0.651, 1.386)	0.783	0.963 (0.626, 1.480)	0.853	0.979 (0.564, 1.699)	0.930
Q3	0.876 (0.5711.344,)	0.532	0.946 (0.579, 1.545)	0.813	0.950 (0.456, 1.979)	0.875
Q4	0.559 (0.361, 0.867)	**0.011**	0.767 (0.466, 1.264)	0.277	0.786 (0.302, 2.050)	0.579
Carbohydrate (gm)	0.998 (0.997, 0.999)	**<0.001**	0.999 (0.998, 1.000)	0.073	0.999 (0.997, 1.001)	0.283
Quartile						
Q1	Reference		Reference		Reference	
Q2	0.712 (0.466, 1.089)	0.113	0.728 (0.465, 1.140)	0.153	0.736 (0.432, 1.251)	0.219
Q3	0.883 (0.622, 1.254)	0.474	1.017 (0.708, 1.461)	0.923	1.034 (0.653, 1.638)	0.871
Q4	0.440 (0.271, 0.715)	**0.002**	0.644 (0.384, 1.081)	0.090	0.672 (0.308, 1.464)	0.273
Total sugars (gm)	0.996 (0.994, 0.998)	**0.002**	0.998 (0.995, 1.000)	0.062	0.998 (0.995, 1.001)	0.110
Quartile						
Q1	Reference		Reference		Reference	
Q2	0.946 (0.553, 1.621)	0.836	0.956 (0.553, 1.652)	0.863	0.963 (0.532, 1.745)	0.888
Q3	0.775 (0.526, 1.142)	0.190	0.874 (0.578, 1.321)	0.498	0.885 (0.549, 1.428)	0.573
Q4	0.437 (0.255, 0.748)	**0.004**	0.561 (0.316, 0.996)	**0.049**	0.577 (0.302, 1.099)	0.085
Dietary fiber (gm)	1.002 (0.987, 1.017)	0.826	1.003 (0.987, 1.020)	0.675	1.006 (0.985, 1.027)	0.555
Quartile						
Q1	Reference		Reference		Reference	
Q2	0.734 (0.500, 1.077)	0.110	0.685 (0.447, 1.049)	0.078	0.689 (0.420, 1.129)	0.120
Q3	1.009 (0.675, 1.508)	0.964	0.999 (0.648, 1.541)	0.998	1.010 (0.619, 1.649)	0.962
Q4	0.927 (0.625, 1.375)	0.697	0.940 (0.594, 1.487)	0.779	0.988 (0.546, 1.790)	0.965
Vitamin C (mg)	1.001 (0.999, 1.002)	0.235	0.999 (0.974, 1.026)	0.962	1.001 (1.000, 1.003)	0.118
Quartile						
Q1	Reference		Reference		Reference	
Q2	1.736 (1.190, 2.532)	**0.006**	0.815 (0.511, 1.301)	0.368	1.717 (1.122, 2.628)	**0.019**
Q3	1.495 (1.000, 2.236)	0.050	0.996 (0.527, 1.883)	0.990	1.355 (0.861, 2.132)	0.162
Q4	1.812 (1.204, 2.726)	**0.006**	0.750 (0.524, 1.073)	0.108	1.907 (1.125, 3.233)	**0.022**
Vitamin D (D2 + D3) (mcg)	0.994 (0.971, 1.018)	0.596	0.998 (0.970, 1.026)	0.856	1.002 (0.973, 1.031)	0.891
Quartile						
Q1	Reference		Reference		Reference	
Q2	0.984 (0.621, 1.560)	0.944	0.878 (0.536, 1.437)	0.583	0.907 (0.532, 1.546)	0.684
Q3	1.248 (0.829, 1.879)	0.277	1.268 (0.822, 1.955)	0.262	1.314 (0.806, 2.142)	0.233
Q4	0.889 (0.566, 1.396)	0.599	0.899 (0.550, 1.468)	0.651	0.956 (0.542, 1.686)	0.860
Vitamin E as alpha-tocopherol (mg)	0.991 (0.971, 1.013)	0.411	0.996 (0.971, 1.021)	0.730	0.999 (0.970, 1.028)	0.926
Quartile						
Q1	Reference		Reference		Reference	
Q2	0.904 (0.567, 1.439)	0.659	0.860 (0.541, 1.369)	0.502	0.843 (0.493, 1.441)	0.483
Q3	0.698 (0.469, 1.041)	0.076	0.669 (0.420, 1.065)	0.086	0.664 (0.384, 1.148)	0.123
Q4	0.727 (0.504, 1.048)	0.085	0.717 (0.481, 1.070)	0.097	0.701 (0.422, 1.164)	0.145
Magnesium (mg)	0.999 (0.998, 1.000)	0.156	1.000 (0.998, 1.001)	0.446	1.000 (0.998, 1.002)	0.690
Quartile						
Q1	Reference		Reference		Reference	
Q2	0.928 (0.562, 1.532)	0.762	0.952 (0.552, 1.643)	0.852	0.955 (0.496, 1.838)	0.875
Q3	1.040 (0.702, 1.540)	0.839	1.029 (0.630, 1.681)	0.902	1.030 (0.556, 1.907)	0.914
Q4	0.750 (0.443, 1.270)	0.274	0.846 (0.480, 1.492)	0.542	0.888 (0.386, 2.041)	0.751
Zinc (mg)	0.984 (0.961, 1.008)	0.194	0.999 (0.974, 1.026)	0.962	1.008 (0.966, 1.051)	0.694
Quartile						
Q1	Reference		Reference		Reference	
Q2	0.823 (0.528, 1.283)	0.378	0.815 (0.511, 1.301)	0.368	0.822 (0.468, 1.441)	0.444
Q3	0.926 (0.534, 1.606)	0.777	0.996 (0.527, 1.883)	0.990	0.990 (0.434, 2.261)	0.979
Q4	0.611 (0.420, 0.889)	**0.012**	0.750 (0.524, 1.073)	0.108	0.774 (0.432, 1.389)	0.342

Logistic regression models:

Model 1: no covariates were adjusted.

Model 2 was adjusted for sex, age, race, education level, marital status, and ratio of family income to poverty.

Model 3 was adjusted for sex, age, race, education level, marital status, ratio of family income to poverty, smoking status, total cholesterol, direct HDL-cholesterol, selenium, urinary iodine, and alcohol consumption status.

Bold values represent statistical significance.

OR, odds ratio; CI, confidence interval; PUFA, polyunsaturated fatty acid.

**Figure 2:**
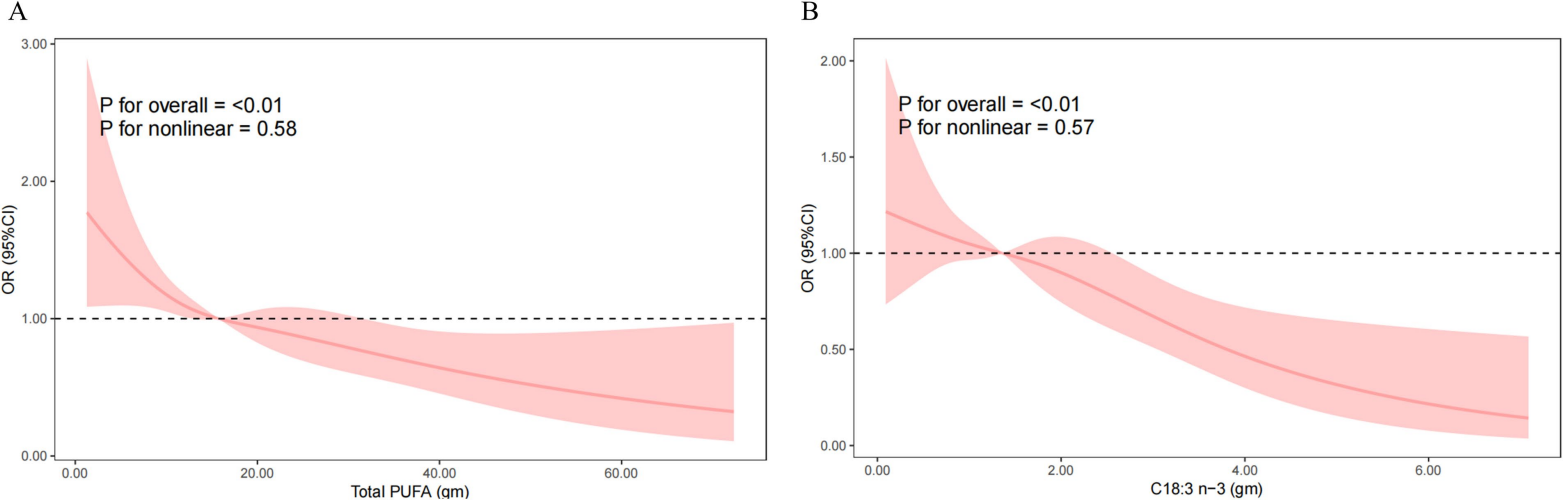
Nonlinear relationship of total PUFAs intake **(A)** and C18:3 n-3 **(B)** intake with the risk of Hashimoto’s thyroiditis. Model was adjusted for sex, age, race, education level, marital status, ratio of family income to poverty, smoking status, total cholesterol, direct HDL-cholesterol, selenium, urinary iodine, and alcohol consumption status. OR, odds ratio; CI, confidence interval; PUFA, polyunsaturated fatty acid.

In the fully adjusted model (Model 3), each 1 g increase in dietary total PUFAs intake was associated with a 2.2% reduction in HT risk (OR = 0.978, 95% CI: 0.957–1.000, *p* = 0.049), and each 1 g increase in dietary C18:3 n-3 intake was associated with a 21.4% reduction (OR = 0.786, 95% CI: 0.642–0.963, *p* = 0.025). Quartile analyses showed that participants in the fourth quartiles of C18:3 n-3 intake had significantly lower HT risk compared to the lowest quartile (Q4: OR = 0.507, 95% CI: 0.265–0.969, *p* = 0.042). Furthermore, the sensitivity analyses incorporating additional adjustments for hypertension and diabetes yielded consistent results (Supplementary Table 1). Nevertheless, no significant curvilinear association were observed between total PUFAs intake (*p* = 0.58 for non-linearity) or C18:3 n-3 intake (*p* = 0.57 for non-linearity) and HT risk (Figure 2).

### 3.4 Subgroup analysis of the association between total PUFA and C18:3 n-3 intake and HT risk

**Figure 3:**
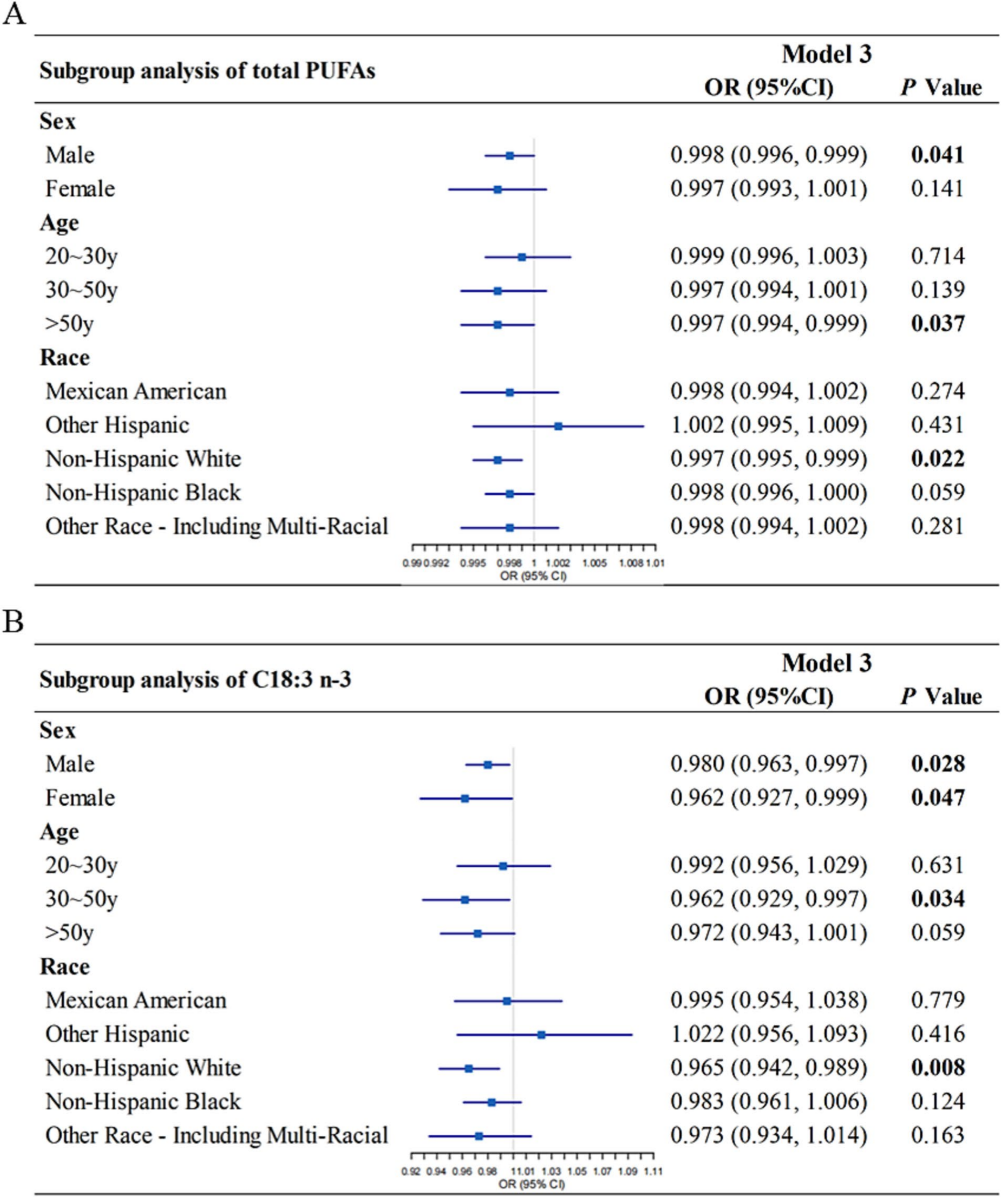
Subgroup analysis of the association between dietary total PUFAs intake **(A)**, C18:3 n-3 **(B)** intake and Hashimoto’s thyroiditis, stratified by sex, age, race. Model 3 was adjusted for gender, age, race, education level, marital status, ratio of family income to poverty, smoking status, total cholesterol, direct HDL-cholesterol, selenium, urinary iodine, and alcohol consumption status. OR, odds ratio; CI, confidence interval; PUFA, polyunsaturated fatty acid.

To evaluate the consistency of associations across population subgroups, subgroup analyses were performed based on age, sex, and race. Dietary total PUFAs intake was significantly and inversely associated with HT risk in males (Model 3: OR = 0.998, 95% CI: 0.996–0.999, *p* = 0.041), individuals aged > 50 years (Model 3: OR = 0.997, 95% CI 0.994–0.999, *p* = 0.037) and non-Hispanic White individuals (Model 3: OR = 0.997, 95% CI 0.995–0.999, *p* = 0.022) (Figure 3A). The inverse association between C18:3 n-3 intake and HT remained significant in several strata. Specifically, C18:3 n-3 intake was inversely associated with HT in males (Model 3: OR = 0.980, 95% CI: 0.963–0.997, *p* = 0.028), females (Model 3: OR = 0.962, 95% CI: 0.927–0.999, *p* = 0.047), individuals aged 30–50 years (Model 3: OR = 0.962, 95% CI 0.929–0.997, *p* = 0.034) and non-Hispanic White individuals (Model 3: OR = 0.965, 95% CI 0.942–0.989, *p* = 0.008) (Figure 3B).

To explore whether differences in HT risk across demographic groups could be attributed to variation in dietary C18:3 n-3 intake, we compared the intake of C18:3 n-3 across sex, age and race (Table 5). The results revealed that males had significantly higher C18:3 intake than females (Kruskal-Wallis test: *p* < 0.001; logistic regression: OR = 0.748, 95% CI: 0.692–0.809, *p* < 0.001). Given that females exhibited higher HT risk in univariate analysis, this finding further supported that insufficient intake of C18:3 n-3 may contribute to HT development.

**Table 5:** Distribution and logistic regression analysis of C18:3 n-3 intake levels by sex, age and race.

Characteristic	C18:3 n-3	*p* value^1^	OR (95%CI)^2^	*p* value^3^
Sex		**<0.001**		
Male	1.880 (1.275)		Reference	
Female	1.443 (0.993)		0.748 (0.692,0.809)	**<0.001**
Age		0.946		
20 ~ 30y	1.695 (1.220)		Reference	
30 ~ 50y	1.648 (1.136)		0.973 (0.884,1.071)	0.558
>50y	1.639 (1.147)		0.981 (0.867,1.110)	0.744
Race		0.560		
Mexican American	1.547 (0.996)		Reference	
Other Hispanic	1.713 (1.371)		1.049 (0.901,1.221)	0.517
Non-Hispanic White	1.666 (1.132)		1.025 (0.932,1.127)	0.591
Non-Hispanic Black	1.695 (1.348)		1.016 (0.916,1.128)	0.743
Other Race - Including Multi-Racial	1.546 (1.112)		0.917 (0.786,1.069)	0.248

1 Pearson’s X^2^: Rao & Scott adjustment; Design-based Kruskal-Wallis test.

2 The box-cox transformation was applied to C18:3 to make it normal distributed.

3 Logistic regression model was adjusted for sex, age, race, education level, marital status, ratio of family income to poverty, smoking status, total cholesterol, direct HDL-cholesterol, selenium, urinary iodine, and alcohol consumption status.

Bold values represent statistical significance.

OR, odds ratio; CI, confidence interval.

### 3.5 Demographic and clinical characteristics of case-control participants

Given that the NHANES-based analysis revealed a significant inverse association between dietary polyunsaturated fatty acids (particularly C18:3 n-3) and the risk of HT, a case-control study was conducted to validate these findings. The study included 125 HT patients and 57 healthy controls. No significant differences were observed in sex, age, or BMI between groups (*p* > 0.05), supporting the comparability of the matched design (Table 6). However, compared to the healthy control group, the HT group had a significantly higher proportion of participants with less than a college education, those who were married, and those with a family history of HT (*p* < 0.05).

**Table 6:** Demographic and clinical characteristics of participants in the case-control study.

Characteristic	Hashimoto’s thyroiditis group (*n* = 125)^1^	Control group (*n* = 57)^1^	*p* value^2^
Sex (%)			0.586
Male	18 (14.4%)	10 (17.5%)	
Female	107 (85.6%)	47 (82.5%)	
Age (years)	32.68 (10.10)	30.10 (7.36)	0.213
BMI (kg/m^2^)	21.85 (2.95)	22.06 (2.39)	0.731
Education level (%)			**0.039**
Less than undergraduate degree	19 (15.2%)	2 (3.5%)	
Undergraduate degree	44 (35.2%)	18 (31.6%)	
Master’s degree or above	62 (49.6%)	37 (64.9%)	
Marital status (%)			**<0.001**
Married	70 (56%)	15 (26.32%)	
Unmarried/widowed	55 (44%)	42 (73.68%)	
Smoking (%)			0.999
Frequent	0	0	
Occasional/never	125 (100%)	57 (100%)	
Drinking (%)			0.773
Frequent	15 (12%)	6 (10.53%)	
Occasional/never	110 (88%)	51 (89.47%)	
Family history of HT (%)			**0.001**
Yes	23 (18.4%)	0	
No	102 (81.6%)	57 (100%)	

1 mean (sd) for continuous; *n* (%) for categorical.

2 Pearson’s X^2^: Rao & Scott adjustment; Design-based Kruskal-Wallis test.

Bold values represent statistical significance.

### 3.6 Erythrocyte phospholipid PUFA composition

As shown in Table 7, erythrocyte levels of total PUFAs (35.075 ± 6.760% vs. 39.867 ± 9.905%, *p* = 0.041), total n-3 PUFAs (3.277 ± 1.720% vs. 5.516 ± 6.613%, *p* = 0.038), EPA (C20:5 n-3; 0.635 ± 0.167% vs. 0.891 ± 0.289%, *p* = 0.048), DHA (C22:6 n-3; 1.428 ± 1.190% vs. 2.272 ± 0.974%, *p* = 0.006), and the n-3 index (2.116 ± 0.913% vs. 3.123 ± 0.821%, *p* = 0.033) were significantly lower in HT patients than in healthy controls, suggesting a possible link between systemic n-3 PUFA deficiency and HT pathogenesis.

**Table 7:** Composition of erythrocyte phospholipid PUFAs in case-control participants.

Fatty acid	Hashimoto’s thyroiditis group (*n* = 125)^1^	Control group (*n* = 57)^1^	*p* value^2^
Total PUFAs (%)	35.075 (6.760)	39.867 (9.905)	**0.041**
n-6 PUFA	32.159 (6.807)	34.954 (4.447)	0.263
C18:2 n-6	1.806 (7.337)	0.397 (1.902)	0.502
C20:3 n-6	0.841 (0.680)	0.955 (0.512)	0.497
C20:4 n-6	12.364 (6.808)	12.321 (8.501)	0.705
n-3 PUFA	3.277 (1.720)	5.516 (6.613)	**0.038**
C18:3 n-3	1.290 (1.578)	2.210 (3.077)	0.079
C20:5 n-3	0.635 (0.167)	0.891 (0.289)	**0.048**
C22:6 n-3	1.428 (1.190)	2.272 (0.974)	**0.006**
n-3 index	2.116 (0.913)	3.123 (0.821)	**0.033**

1 mean (sd) for continuous; *n* (%) for categorical.

2 Pearson’s X^2^: Rao & Scott adjustment; Design-based Kruskal-Wallis test.

Bold values represent statistical significance.

PUFA, polyunsaturated fatty acid.

### 3.7 Associations between erythrocyte phospholipid PUFA levels and HT risk

Adjusted associations between erythrocyte phospholipid PUFA levels and HT risk are presented in Table 8. In the unadjusted model (Model 1), higher levels of total n-3 PUFAs, C18:3 n-3, EPA, DHA and the n-3 index were significantly associated with reduced HT risk (*p* < 0.05). In both Model 2 and Model 3, the inverse associations of total n-3 PUFAs (Model 2: OR = 0.571, 95% CI: 0.322–0.987, *p* = 0.039; Model 3: OR = 0.616, 95% CI: 0.443–0.992, *p* = 0.041), C18:3 n-3 (Model 2: OR = 0.597, 95% CI: 0.465–0.823, *p* = 0.034; Model 3: OR = 0.627, 95% CI: 0.558–0.874, *p* = 0.037), EPA (C20:5 n-3: Model 2: OR = 0.651, 95% CI: 0.412–0.964, *p* = 0.011; Model 3: OR = 0.736, 95% CI: 0.581–0.975, *p* = 0.042), and the n-3 index (Model 2: OR = 0.674, 95% CI: 0.525–0.973, *p* = 0.028; Model 3: OR = 0.790, 95% CI: 0.531–0.984, *p* = 0.046) with HT risk remained statistically significant.

**Table 8:** Associations between erythrocyte phospholipid PUFA levels and the risk of Hashimoto’s thyroiditis.

Fatty acids	Model 1 OR (95% CI)	*p* value	Model 2 OR (95% CI)	*p* value	Model 3 OR (95% CI)	*p* value
Total n-3 PUFAs	0.514 (0.312–0.974)	**0.027**	0.571 (0.322–0.987)	**0.039**	0.616 (0.443–0.992)	**0.041**
C18:3 n-3	0.432 (0.386–0.673)	**0.016**	0.597 (0.465–0.823)	**0.034**	0.627 (0.558–0.874)	**0.037**
C20:5 n-3	0.502 (0.374–0.858)	**0.032**	0.651 (0.412–0.964)	**0.011**	0.736 (0.581–0.975)	**0.042**
C22:6 n-3	0.618 (0.426–0.971)	**0.049**	0.735 (0.593–1.011)	0.271	0.844 (0.613–1.120)	0.345
n-3 index	0.534 (0.412–0.936)	**0.014**	0.674 (0.525–0.973)	**0.028**	0.790 (0.531–0.984)	**0.046**

Logistic regression models:

Model 1: no covariates were adjusted.

Model 2 was adjusted for sex, and age.

Model 3 was adjusted for sex, age, marital status, family history of HT, body mass index, educational level, total erythrocyte n-6 PUFA, and total saturated fatty acids.

Bold values represent statistical significance.

OR, odds ratio; CI, confidence interval; PUFA, polyunsaturated fatty acid.

## 4 Discussion

To our knowledge, this is the first study to combine a cross-sectional analysis of NHANES data with a population-based case-control validation to investigate the relationship between dietary nutrient intake and the risk of HT. The NHANES analysis revealed no significant associations between HT risk and the intake of protein, carbohydrates, total sugars, dietary fiber, vitamin C, vitamin D (including D2 and D3), vitamin E (*α*-tocopherol), magnesium, or zinc. However, a significant inverse association was observed between energy, total fat, n-3 PUFAs, particularly α-linolenic acid (C18:3 n-3), and HT risk. These findings about PUFAs were confirmed by the case-control study, which demonstrated significantly lower erythrocyte membrane levels of total n-3 PUFAs, EPA, DHA, and the n-3 index in HT patients compared to healthy controls.

HT is an autoimmune disease with a strong genetic basis, triggered by environmental factors that lead to immune dysregulation and a loss of self-tolerance (18). Among modifiable environmental factors, dietary patterns and nutrient intake play critical roles. A study of Polish HT patients revealed insufficient intake of PUFA-rich foods (19), aligning with our findings that lower n-3 PUFA intake is associated with increased HT risk.

The Mediterranean diet (MD), known for its antioxidants and anti-inflammatory components, has been shown to improve inflammatory status in various autoimmune and metabolic diseases (20). N-3 PUFAs are one of the key components of MD, with established roles in modulating immune function and reducing systemic inflammation (21). In patients with hyperthyroidism, adherence to the MD has been associated with reductions in oxidative stress markers, highlighting its relevance to thyroid health (22). The beneficial effects of MD have been partly attributed to its high content of monounsaturated fatty acids, a favorable n-3/n-6 PUFA ratio, and low saturated fat content (23–25). Furthermore, an anti-inflammatory diet enriched in n-3 PUFAs has been shown to reduce thyroid autoantibody levels (21). These findings support that increasing n-3 PUFA intake may help mitigate autoimmune thyroid inflammation and reduce HT risk.

Rich dietary sources of n-3 PUFAs include fatty fish such as salmon, mackerel, sardines, and anchovies, which are high in EPA and DHA. In contrast, plant-based sources such as flaxseeds, chia seeds, walnuts, and canola oil are rich in *α*-linolenic acid, which can be endogenously converted to EPA and DHA, although this conversion is relatively inefficient in humans. Incorporating both marine and plant-based n-3 PUFA sources into the diet may therefore offer complementary benefits and represent an accessible strategy to improve n-3 PUFA status and potentially reduce HT risk.

To further investigate whether sex differences in HT risk could be attributed to variations in n-3 PUFA intake, we analyzed the intake of C18:3 n-3 across demographic subgroups. The results revealed that C18:3 n-3 intake was significantly higher in males than in females, a trend supported by findings from Italy and New Zealand, where males consumed substantially more PUFA than females (8 vs. 6 g/day, *p* < 0.001; 13.1 vs. 9.6 g/day) (26, 27). These differences, likely stemming from sex-based dietary habits (24), may partially explain the higher prevalence of HT among women observed in our study.

Part of the lower dietary intake observed in the HT group may also be attributable to the higher proportion of women, who generally consume less energy and nutrients than men. Importantly, after adjustment for sex in the multivariable models, the associations between dietary n-3 PUFA intake and HT remained statistically significant, suggesting that sex distribution alone does not fully account for the observed findings. Furthermore, in our case-control study, n-3 PUFA was expressed as a percentage of total fatty acids rather than as absolute intake, which is not affected by sex differences in total food or energy consumption. The consistency of findings across both absolute intake (NHANES) and proportional composition (case-control study) therefore strengthens the robustness of the observed association.

The n-3 index, a validate biomarker representing the combined percentage of EPA and DHA in erythrocyte membranes, was proposed by Harris and von Schacky in 2004 as a reflection of long-term n-3 PUFAs status (28). In our case-control study, the n-3 index was significantly lower in HT patients and inversely associated with HT prevalence, further supporting the relevance of n-3 PUFA status to autoimmune thyroid disease.

The potential protective mechanism of n-3 PUFAs against HT may involve immunomodulatory effects and the suppression of pro-inflammatory cytokines (29). N-3 PUFAs reduce the synthesis of pro-inflammatory mediators such as prostaglandin E2 and leukotriene B4 by competitively inhibiting the metabolism of arachidonic acid, while simultaneously promoting the production of anti-inflammatory mediators including resolvins and protectins (30). This shift in lipid mediator production may alleviate chronic inflammation and attenuate autoimmune responses in HT. Additionally, interleukin-1β (IL-1β), a known driver of thyroid cell apoptosis (31), can be downregulated by dietary n-3 PUFAs, thereby reducing thyroid tissue damage (32).

This study has several strengths. It combined a nationally representative NHANES dataset with an independent case-control study, allowing for cross-validation of findings. Rigorous multivariable regression modeling was employed to adjust for a wide range of potential confounders, minimizing the risk of residual bias. Additionally, stratified analyses by sex, age, and race provided nuanced insights into subgroup-specific associations. Importantly, the case-control component incorporated erythrocyte n-3 index as a robust biomarker of long-term n-3 PUFA status. Compared to self-reported intake or serum phospholipid levels, the n-3 index offers superior biological stability and temporal relevance, strengthening the observed association between n-3 PUFA exposure and HT risk.

Nonetheless, several limitations should be acknowledged. First, the cross-sectional nature of NHANES data and the observational design of the case-control study preclude conclusions about causality. Second, although TPOAb/TGAb measurement and dietary intake data were collected during the same assessment, and participants were informed of their thyroid-related laboratory results after all baseline data collection had been completed, we were unable to exclude the possibility that some individuals might have already been aware of their HT status prior to the survey. Such awareness could potentially have led to dietary modification. However, as clinical recommendations for HT generally encourage increasing dietary sources of n-3 PUFAs, any such behavior change would more likely bias the observed association toward the null. Therefore, the lower n-3 PUFA intake observed among HT participants in our study may represent a conservative estimate of the true association. Third, despite adjusting for numerous confounders, residual confounding from unmeasured factors cannot be completely excluded. Third, NHANES dietary intake data were derived from a 24-h recall, which may be subject to recall bias and may not accurately reflect habitual intake.

In conclusion, our findings demonstrate a significant inverse association between n-3 PUFA intake, particularly *α*-linolenic acid, EPA, and n-3 index, and the risk of HT. These results suggest that insufficient n-3 PUFA consumption may be a modifiable risk factor for HT. Further longitudinal and interventional studies are warranted to determine the optimal type, dose, and duration of n-3 PUFA supplementation in the prevention and management of autoimmune thyroid diseases. These insights may inform future dietary guidelines and public health strategies aimed at reducing the burden of HT.

## Data Availability

The original contributions presented in the study are included in the article/Supplementary material, further inquiries can be directed to the corresponding authors.
