# Supplementary material for: Investigating the associations between dietary nutrient intake and risk of Hashimoto’s thyroiditis: a cross-sectional study from NHANES and a case-control study
Source: Front Nutr. 2025 Dec 31;12:1731662. doi: 10.3389/fnut.2025.1731662 (PMC12801514; doi:10.3389/fnut.2025.1731662)
Supplement: Supplementary file 1 [file Table_1.DOCX]

| **Supplementary Table 1.** Sensitivity analyses of the associations between dietary nutrient intake and the risk of Hashimoto's thyroiditis. | | | | |
| --- | --- | --- | --- | --- |
| **Characteristic** | **Model 3 +Diabetes status** | | **Model 3 +Hypertension status** | |
|  | **OR (95%CI)** | ***P* Value** | **OR (95%CI)** | ***P* Value** |
| **Energy (kcal)** | 0.99965  (0.99934, 0.99997) | **0.037** | 0.99964  (0.99934, 0.99995) | **0.027** |
| Quartile |  |  |  |  |
| Q1 | Reference |  | Reference |  |
| Q2 | 0.767 (0.461, 1.277) | 0.251 | 0.736 (0.448, 1.207) | 0.186 |
| Q3 | 0.861 (0.488, 1.520) | 0.544 | 0.822 (0.488, 1.384) | 0.404 |
| Q4 | 0.498 (0.237, 1.048) | 0.062 | 0.477 (0.239, 0.952) | **0.039** |
| **Total fat (gm)** | 0.990 (0.985, 0.996) | **0.003** | 0.990 (0.985, 0.996) | **0.003** |
| Quartile |  |  |  |  |
| Q1 | Reference |  | Reference |  |
| Q2 | 0.587 (0.349, 0.988) | **0.046** | 0.576 (0.351, 0.945) | **0.034** |
| Q3 | 0.475 (0.250, 0.901) | **0.029** | 0.467 (0.250, 0.872) | **0.024** |
| Q4 | 0.373 (0.205, 0.676) | **0.007** | 0.363 (0.203, 0.650) | **0.005** |
| **Total PUFAs (gm)** | 0.978 (0.957, 1.000) | 0.053 | 0.978 (0.957, 1.000) | **0.049** |
| Quartile |  |  |  |  |
| Q1 | Reference |  | Reference |  |
| Q2 | 0.651 (0.371, 1.140) | 0.110 | 0.635 (0.371, 1.086) | 0.086 |
| Q3 | 0.636 (0.341, 1.186) | 0.126 | 0.627 (0.346, 1.139) | 0.107 |
| Q4 | 0.495 (0.236, 1.037) | 0.059 | 0.492 (0.240, 1.009) | 0.052 |
| **C18:2 n-6 (gm)** | 0.977 (0.953, 1.002) | 0.068 | 0.977 (0.954, 1.002) | 0.064 |
| Quartile |  |  |  |  |
| Q1 | Reference |  | Reference |  |
| Q2 | 0.618 (0.331, 1.156) | 0.109 | 0.602 (0.328, 1.107) | 0.090 |
| Q3 | 0.653 (0.355, 0.355) | 0.138 | 0.646 (0.357, 1.170) | 0.126 |
| Q4 | 0.516 (0.255, 1.045) | 0.062 | 0.514 (0.259, 1.019) | 0.055 |
| **C18:3 n-3 (gm)** | 0.785 (0.636, 0.970) | **0.030** | 0.785 (0.638, 0.966) | **0.027** |
| Quartile |  |  |  |  |
| Q1 | Reference |  | Reference |  |
| Q2 | 0.740 (0.418, 1.311) | 0.246 | 0.739 (0.426, 1.284) | 0.237 |
| Q3 | 0.838 (0.429, 1.639) | 0.543 | 0.834 (0.434, 1.604) | 0.532 |
| Q4 | 0.504 (0.255, 0.994) | **0.049** | 0.505 (0.259, 0.986) | **0.046** |
| **C18:4 n-3 (gm)** | 3.346 (0.025, 450.665) | 0.586 | 3.120 (0.021, 457.059) | 0.618 |
| Quartile |  |  |  |  |
| Q1 | Reference |  | Reference |  |
| Q2 | 0.879 (0.506, 1.528) | 0.589 | 0.897 (0.523, 1.536) | 0.647 |
| Q3 | 0.870 (0.524, 1.446) | 0.528 | 0.865 (0.528, 1.417) | 0.510 |
| Q4 | 1.169 (0.685, 1.996) | 0.501 | 1.158 (0.694, 1.932) | 0.520 |
| **C20:4 n-6 (gm)** | 0.179 (0.026, 1.229) | 0.073 | 0.182 (0.028, 1.180) | 0.069 |
| Quartile |  |  |  |  |
| Q1 | Reference |  | Reference |  |
| Q2 | 0.759 (0.393, 1.467) | 0.345 | 0.759 (0.399, 1.443) | 0.344 |
| Q3 | 0.613 (0.314, 1.199) | 0.125 | 0.621 (0.331, 1.163) | 0.116 |
| Q4 | 0.608 (0.307, 1.206) | 0.126 | 0.603 (0.310, 1.174) | 0.116 |
| **C20:5 n-3 (gm)** | 2.590 (0.490, 13.686) | 0.224 | 2.419 (0.461, 12.699) | 0.259 |
| Quartile |  |  |  |  |
| Q1 | Reference |  | Reference |  |
| Q2 | 0.921 (0.468, 1.811) | 0.776 | 0.938 (0.487, 1.804) | 0.823 |
| Q3 | 0.681 (0.375, 1.236) | 0.166 | 0.684 (0.383, 1.222) | 0.166 |
| Q4 | 0.754 (0.430, 1.324) | 0.266 | 0.740 (0.428, 1.281) | 0.236 |
| **C22:5 n-3 (gm)** | 0.041 (0.000, 33.908) | 0.305 | 0.033 (0.000, 29.704) | 0.286 |
| Quartile |  |  |  |  |
| Q1 | Reference |  | Reference |  |
| Q2 | 0.902 (0.506, 1.608) | 0.676 | 0.901 (0.515, 1.575) | 0.673 |
| Q3 | 0.595 (0.318, 1.114) | 0.089 | 0.598 (0.328, 1.092) | 0.083 |
| Q4 | 0.605 (0.318, 1.151) | 0.104 | 0.598 (0.323, 1.107) | 0.089 |
| **C22:6 n-3 (gm)** | 1.500 (0.543, 4.145) | 0.384 | 1.437 (0.521, 3.962) | 0.439 |
| Quartile |  |  |  |  |
| Q1 | Reference |  | Reference |  |
| Q2 | 0.639 (0.371, 1.100) | 0.090 | 0.641 (0.379, 1.084) | 0.086 |
| Q3 | 0.683 (0.358, 1.304) | 0.200 | 0.686 (0.371, 1.271) | 0.192 |
| Q4 | 0.847 (0.464, 1.550) | 0.527 | 0.824 (0.463, 1.467) | 0.454 |
| **Protein (gm)** | 0.993 (0.980, 1.007) | 0.294 | 0.993 (0.980, 1.007) | 0.278 |
| Quartile |  |  |  |  |
| Q1 | Reference |  | Reference |  |
| Q2 | 0.978 (0.549, 1.745) | 0.929 | 0.975 (0.555, 1.713) | 0.918 |
| Q3 | 0.966 (0.438, 2.130) | 0.919 | 0.944 (0.448, 1.988) | 0.859 |
| Q4 | 0.795 (0.283, 2.235) | 0.607 | 0.785 (0.295, 2.089) | 0.577 |
| **Carbohydrate (gm)** | 0.999 (0.997, 1.001) | 0.331 | 0.999 (0.997, 1.001) | 0.263 |
| Quartile |  |  |  |  |
| Q1 | Reference |  | Reference |  |
| Q2 | 0.749 (0.427, 1.314) | 0.255 | 0.731 (0.424, 1.261) | 0.216 |
| Q3 | 1.072 (0.641, 1.792) | 0.753 | 1.023 (0.641, 1.634) | 0.911 |
| Q4 | 0.682 (0.295, 1.574) | 0.306 | 0.667 (0.302, 1.472) | 0.266 |
| **Total sugars (gm)** | 0.998 (0.995, 1.001) | 0.148 | 0.998 (0.995, 1.001) | 0.106 |
| Quartile |  |  |  |  |
| Q1 | Reference |  | Reference |  |
| Q2 | 0.966 (0.512, 1.821) | 0.898 | 0.954 (0.519, 1.755) | 0.861 |
| Q3 | 0.907 (0.543, 1.513) | 0.656 | 0.877 (0.542, 1.420) | 0.541 |
| Q4 | 0.591 (0.294, 1.188) | 0.115 | 0.571 (0.298, 1.095) | 0.081 |
| **Dietary fiber (gm)** | 1.006 (0.984, 1.028) | 0.551 | 1.005 (0.985, 1.027) | 0.568 |
| Quartile |  |  |  |  |
| Q1 | Reference |  | Reference |  |
| Q2 | 0.701 (0.414, 1.188) | 0.150 | 0.682 (0.415, 1.122) | 0.112 |
| Q3 | 1.017 (0.600, 1.724) | 0.939 | 1.000 (0.613, 1.634) | 0.998 |
| Q4 | 1.006 (0.536, 1.887) | 0.983 | 0.977 (0.539, 1.769) | 0.928 |
| **Vitamin C (mg)** | 1.001 (1.000, 1.003) | 0.117 | 1.001 (1.000, 1.003) | 0.122 |
| Quartile |  |  |  |  |
| Q1 | Reference |  | Reference |  |
| Q2 | 1.735 (1.091, 2.760) | **0.027** | 1.710 (1.109, 2.637) | **0.022** |
| Q3 | 1.365 (0.837, 2.226) | 0.171 | 1.352 (0.845, 2.163) | 0.173 |
| Q4 | 1.947 (1.107, 3.424) | **0.028** | 1.906 (1.108, 3.278) | **0.026** |
| **Vitamin D (D2 + D3) (mcg)** | 1.002 (0.973, 1.032) | 0.851 | 1.001 (0.972, 1.031) | 0.931 |
| Quartile |  |  |  |  |
| Q1 | Reference |  | Reference |  |
| Q2 | 0.910 (0.520, 1.593) | 0.696 | 0.907 (0.525, 1.568) | 0.686 |
| Q3 | 1.347 (0.804, 2.258) | 0.208 | 1.313 (0.797, 2.164) | 0.238 |
| Q4 | 0.971 (0.530, 1.779) | 0.908 | 0.943 (0.528, 1.684) | 0.819 |
| **Vitamin E as alpha-tocopherol (mg)** | 0.999 (0.969, 1.029) | 0.919 | 0.998 (0.969, 1.028) | 0.909 |
| Quartile |  |  |  |  |
| Q1 | Reference |  | Reference |  |
| Q2 | 0.856 (0.480, 1.524) | 0.533 | 0.840 (0.486, 1.452) | 0.475 |
| Q3 | 0.665 (0.376, 1.177) | 0.131 | 0.656 (0.379, 1.135) | 0.112 |
| Q4 | 0.720 (0.413, 1.253) | 0.197 | 0.695 (0.413, 1.168) | 0.141 |
| **Magnesium (mg)** | 1.000 (0.998, 1.002) | 0.686 | 1.000 (0.998, 1.002) | 0.667 |
| Quartile |  |  |  |  |
| Q1 | Reference |  | Reference |  |
| Q2 | 0.963 (0.484, 1.915) | 0.897 | 0.944 (0.488, 1.826) | 0.841 |
| Q3 | 1.042 (0.549, 1.977) | 0.881 | 1.019 (0.547, 1.900) | 0.944 |
| Q4 | 0.894 (0.378, 2.116) | 0.762 | 0.875 (0.380, 2.012) | 0.716 |
| **Zinc (mg)** | 1.007 (0.964, 1.052) | 0.720 | 1.007 (0.966, 1.050) | 0.699 |
| Quartile |  |  |  |  |
| Q1 | Reference |  | Reference |  |
| Q2 | 0.813 (0.452, 1.462) | 0.421 | 0.819 (0.462, 1.453) | 0.438 |
| Q3 | 1.010 (0.417, 2.446) | 0.979 | 0.985 (0.427, 2.272) | 0.968 |
| Q4 | 0.770 (0.409, 1.450) | 0.351 | 0.772 (0.428, 1.394) | 0.335 |
| Logistic regression models: | | | | |
| Model 3 was adjusted for sex, age, race, education level, marital status, ratio of family income to poverty, total cholesterol, direct HDL-cholesterol, smoking status, selenium, urinary iodine, and alcohol consumption status. | | | | |
| Bold values represent statistical significance.  Abbreviations: OR, odds ratio; CI, confidence interval; PUFA, polyunsaturated fatty acid. | | | | |
